# Comparative effectiveness of three treatment options for slade and dodds grade III-IV scaphoid nonunion: a retrospective study

**DOI:** 10.1186/s12891-023-06320-1

**Published:** 2023-03-17

**Authors:** Zhenye Zhong, Meiyang Wei, Zhaoying Jiang, Jinshui Chen, Yanda He, Kaifeng Lin

**Affiliations:** 1 Department of Orthopedics, 900th Hospital of the Joint Logistic Support Force, 156 West 2nd Ring Road North, Fuzhou, Fujian China; 2 Department of Orthopedics, Shengli Clinical Medical College of Fujian Medical University, 134 East Street, Fuzhou, Fujian China; 3 Department of Obstetrics and Gynecology, The Fourth Affiliated Hospital, International Institutes of Medicine, Zhejiang University School of Medicine, Yiwu, Zhejiang China; 4 Department of Orthopedics, Hui ’an County Hospital, 184 Zhongshan North Street, Quanzhou, Fujian China; 5 Department of Orthopedics, Fujian provincial hospital, 134 East Street, Fuzhou, Fujian China

**Keywords:** Scaphoid, Nonunion, Percutaneous fixation, PRP

## Abstract

**Objective:**

To compare the clinical efficacy of open debridement screw fixation combined with bone grafting, percutaneous screw fixation, and percutaneous screw fixation combined with injection of platelet-rich plasma (PRP) for the treatment of Slade and Dodds Grade III to IV scaphoid nonunion (SNU).

**Methods:**

This retrospective study included patients with Grade III (25 patients) and Grade IV (28 patients) SNU. They were treated with open surgery bone grafting and internal fixation (group A), percutaneous screw fixation (group B) or percutaneous screw fixation and PRP injection (group C) from January 2015 to May 2020. The fracture consolidation rate, VAS score, and Mayo wrist function score were compared across the three groups.

**Results:**

The consolidation rate was not significantly different among the three groups for both Grade III and IV SNU. However, patients in group C reported significantly less pain and better wrist function 7 days after surgery compared to group A and B, for both nonunion grades. At 3 months after surgery, group C had significantly better VAS and Mayo wrist scores compared to group A for both nonunion grades, and compared to group B for Grade IV SNU. At 6 and 12 months after surgery, patients with Grade IV SNU in groups A and C had significantly better VAS and Mayo wrist scores compared to group B.

**Conclusion:**

This study suggests that percutaneous screw fixation with PRP injection could be a more effective method for treating Grade IV SNU. This approach may reduce postoperative wrist pain and improve wrist function in the early stages after surgery for patients with both Grade III and IV SNU.

**Type of study/level of evidence:**

IV.

**Supplementary Information:**

The online version contains supplementary material available at 10.1186/s12891-023-06320-1.

## Introduction

Scaphoid fracture is the most common fracture of the carpus, particularly among young people. About 5–10% of fractures will finally turn into nonunion [1], causing pain and disability [2]. Slade and Dodds categorized scaphoid nonunion (SNU) into 6 Grades to guide treatment [3]. At present, percutaneous screw fixation strategy is considered to be effective in the treatment of Grade I-III SUN, but cannot be used to treat type Grade VI SNU and its therapeutic effect on Grade IV and V SNU still remains controversial [4–9]. Various treatments are available to improve bone healing and alleviate short-term and long-term symptoms, enabling patients to resume daily activities [1, 10, 11]. PRP has been proved to relieve post-operative pain in scaphoid fracture and improve healing rate of patients with SNU after open surgery [1, 2].

The objectives of this study were to (1) asses the feasibility of percutaneous screw fixation and PRP injection for the treatment of Grade III-IV SNU and (2) investigate the effect of PRP on pain relief and functional recovery in patients with Grade III-IV SNU who received percutaneous screw fixation.

## Methods

### Study Design and setting

This retrospective study was approved by the ethics committee of the 900th Hospital of Joint Logistics Support Force. All patients signed an informed consent form before undergoing surgery. The study enrolled patients who underwent initial surgery for Grade III-IV SNU between January 2015 and May 2020, who were initially misdiagnosed, had a missed diagnosis, or whose initial non-surgical management failed.

The exclusion criteria were: (1) radiographic evidence of osteonecrosis, kyphosis, or displacement; (2) other fractures or dislocation of the wrist; (3) subsequent reoperation within 1 year after the initial surgery, or (4) patients without complete follow-up data.

According to Slade and Dodds SUN classification system [3], two senior doctors assessed the degree of bone absorption at the fracture ends by preoperative CT to classify the Grades, and case would be abandoned when it was in dispute. Based on different surgical treatment methods, patients were divided into three groups for Grade III and Grade IV respectively: open surgery bone grafting and internal fixation (group A), percutaneous screw fixation (group B) and percutaneous screw fixation and PRP injection (group C).

### Surgical techniques

**Fig. 1:**
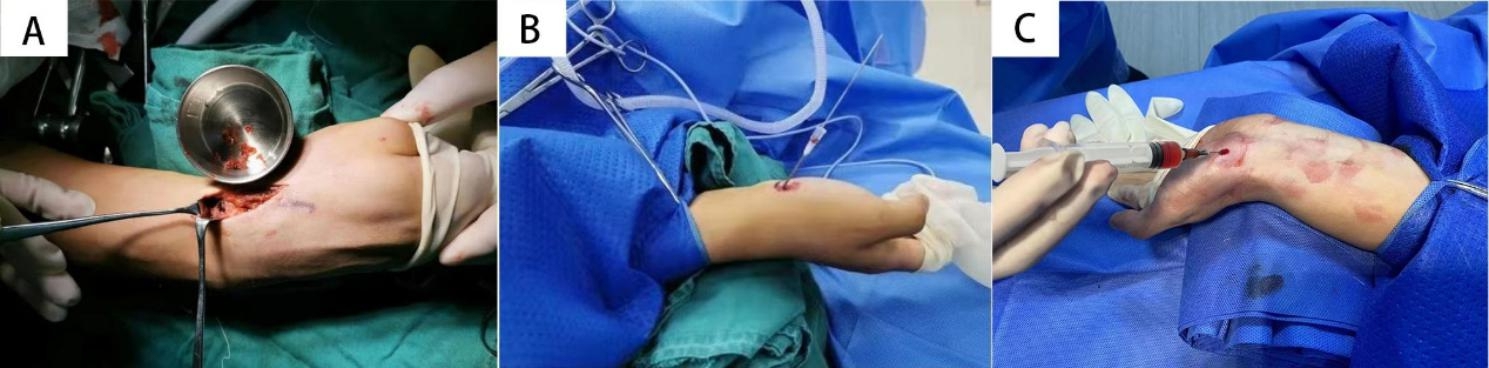
A. Open surgery debridement, bone grafting and internal fixation: Cancellous bone was transplanted onto the fractured ends of the scaphoid. Figure 1B. Percutaneous screw fixation. The gauge syringe needle used as a guide sleeve was inserted into the proximal subchondral bone through the scaphoid tuberosity. Figure 1C. Injection of 1 mL of PRP into the medullary cavity

For open surgery debridement, bone grafting, and internal fixation (group A), the patient was placed in a supine position with the injured limb abducted. After anesthesia, a tourniquet was tied around the upper arm, a radial 3-cm long incision was made on the dorsal wrist at the snuff box. The Dorsal radial carpal ligament and joint capsule were incised longitudinally, the radial nerve and radial artery were carefully protected, and the scaphoid fracture and radial styloid process were exposed. Sclerotic bone was cleared at the fracture ends to open the medullary cavity on both sides until blood exudated from the medullary cavity, i.e., the “red pepper sign”. We removed the styloid process of the radius and sufficient cancellous bone was scraped from the medullary cavity of the distal radius and transplanted to the fracture ends of the scaphoid (Fig. 1A). After reduction, the scaphoid fracture was fixed with a Herbert screw. During surgery, C-arm fluoroscopy was used to ensure that there was no lateral or separation displacement of the fracture and the axis of the wrist was normal. Finally, the joint capsule was sutured and the incision was closed.

For percutaneous screw fixation (group B), the pre-surgical steps were the same as for Group A. The wrist joint was elevated and extended to 90 ° and the thumb and index finger were pulled to achieve closed reduction. If necessary, Kirschner wires were used as joysticks to help reduction. A 12–14 gauge syringe needle used as a guide sleeve was inserted into the proximal subchondral bone through the scaphoid tuberosity (Fig. 1B); C-arm fluoroscopy was performed to confirm that the guide sleeve was located on the long axis of the scaphoid bone. A Kirschner wire was drilled through the guide sleeve and through the fracture line. The cannulated drill was drilled into the bone along the Kirschner wire. If necessary, more Kirschner wire was used to open the medullary cavity at multiple positions in parallel to the direction of the guide sleeve. Then an appropriate length of Herbert screw was inserted along the Kirschner wire. Finally, the Kirschner wire was pulled and the incision was closed.

**Fig. 2:**
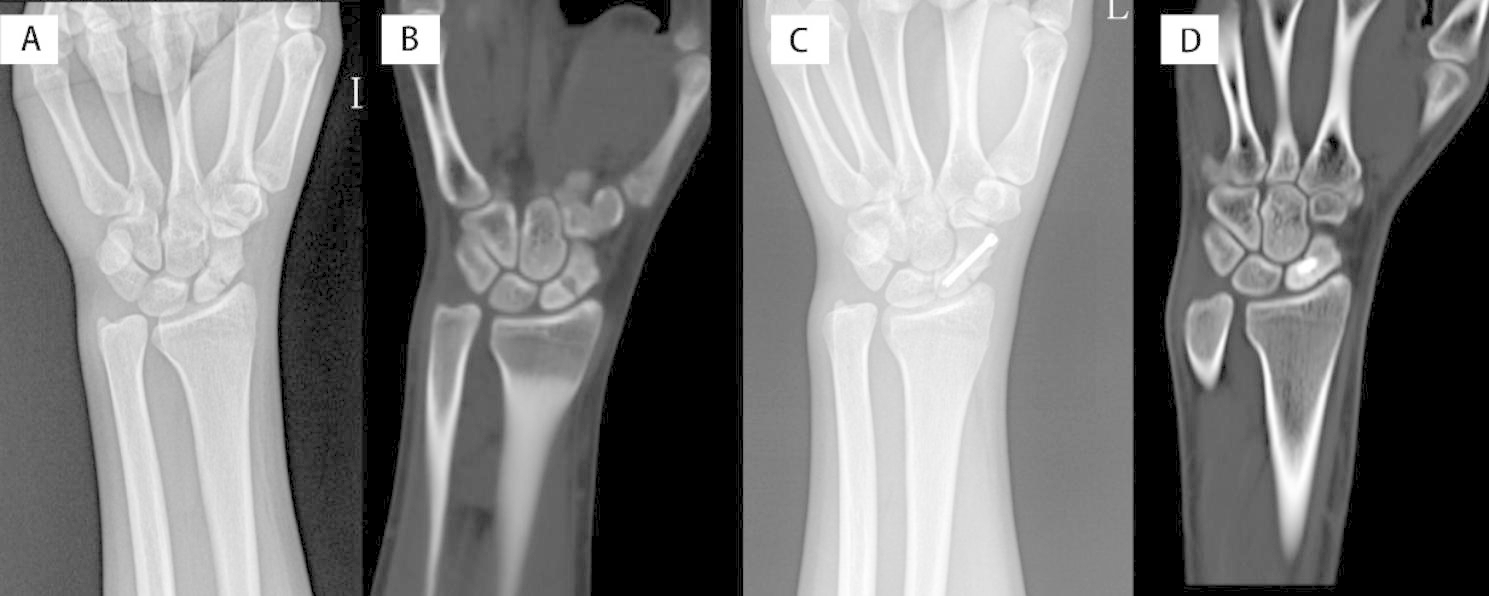
A-B. 19 male patient with scaphoid nonunion in the left hand, with time passed from trauma of 10 months, Preoperative radiographs. Figure 2 C. Immediately radiographs after surgery. Figure 2D. 3 month postoperative radiographs

For percutaneous screw fixation and PRP injection (group C), PRP was prepared using an Arthrex Dual Syringe System (Tehran, Iran). A total of 45 ml of venous blood was draw into a centrifuge Tubes with 5ml of sodium citrate, then it was centrifuged at 1200 rpm for 5 min in the operating room under sterile conditions. The supernatant from the first tube was transferred to the second tube and centrifuged at 2600 rpm for 6 min to obtain 4 mL of PRP. The pre-surgical steps, drilling, placement of the Kirschner wire, and reaming were the same as for Group B. In addition, 1 mL of PRP was injected into the medullary cavity using a syringe before and after the Herbert screw was screwed in (Fig. 1C) and 2 mL of PRP was injected percutaneously into the wrist capsule around the fracture line after the Herbert screw was placed.

### Postoperative management

All patients were treated with the same rehabilitation program after surgery. They were instructed to wear a brace that fixed the wrist joint and proximal interphalangeal joint of the thumb for 4 weeks. The patients were instructed to flex and extend the distal interphalangeal joint of the thumb, while other fingers move freely. Non-weight bearing functional exercises were performed after 2 weeks and passive resistance training was performed under the guidance of rehabilitation therapists or orthopedic surgeons after 4 weeks.

### Clinical and radiological evaluations

X-ray examination was performed on the second day after surgery, and CT examination was performed at 3, 6, and 12 months after surgery until bone healing was observed by imaging (i.e., bone trabeculae were passing through the fracture line) (Fig. 2A-D). Scaphoid union was defined as bone trabeculae passing through the fracture line in CT and absence of clinical manifestation.

The degree of pain and wrist function were assessed using the VAS score (0 to 100) and Mayo wrist function score. The VAS pain score was recorded at 7 days and 3, 6, and 12 months after surgery. The Mayo score of wrist function was evaluated at 3, 6, and 12 months after surgery.

### Statistical analysis

The variables assessed in this retrospective study were the fracture union rate, VAS score, and Mayo wrist function score. The Shapiro-Wilk test indicated the VAS and Mayo wrist function scores were non-normally distributed; these values are presented as median and quartiles and were compared using the Mann Whitney U-test. The dichotomous variable fracture union rate was compared using the Chi-square test. P-values less than 0.05 were considered statistically significant. All data were analyzed using SPSS version 26.0 (SPSS Inc.).

## Results

A total of 53 patients were included in our study, and they were divided into three groups in Grade III and Grade IV, with 11 and 10 patients in Grade III and IV respectively for group A (open surgery bone grafting and internal fixation), 7 and 7 patients in Grade III and IV respectively for group B (percutaneous screw fixation), and 7 and 11 patients in Grade III and IV respectively for group C (percutaneous screw fixation and PRP injection). Of injury reasons, there were 37 patients caused by falling injury, 9 patients caused by heavy objects pressure injury and 7 patients by traffic accident injury. There was no statistically significant difference in the baseline characteristics of the patients with Grade III SNU and Grade IV SNU among the three surgical groups (Table 1). And no patients experienced neurovascular injury during the operation or suffered from any postoperative complications.

**Table 1:** Principal demographic and clinical features of patients studied

Nonunion Grade	Grade III				Grade IV			
Item	Group A	Group B	Group C	*P-*value	Group A	Group B	Group C	*P-*value
Age	23 (19 to 50)	26 (18 to 38)	28 (20 to 46)	0.71	23 (19 to 30)	28 (22 to 38)	24 (18 to 31)	0.06
Sex M/F	10/1	7/0	7/0	0.52	10/0	6/1	9/2	0.38
Injured side L/R	7/4	3/4	3/4	0.59	6/4	3/4	5/6	0.73
Dominance Y/N	9/2	4/3	4/3	0.42	6/4	5/2	6/5	0.77
Time from initial injury to surgery (Mo)	8 (6 to 24)	7 (6 to 16)	8 (6 to 10)	0.48	10 (6 to 15)	9 (6 to 15)	11 (6 to 24)	0.67
Current smoker Y/N	3/8	2/5	2/5	0.99	3/7	2/5	4/7	0.93
Diabetes Y/N	2/9	1/6	2/5	0.78	2/8	1/6	1/10	0.78

Group A, open surgery debridement, bone grafting, and internal fixation; Group B, percutaneous Screw Fixation; Group C,percutaneous screw fixation and PRP injection. M male, F female, L left, R right, Y yes, N no, Mo month

**Fig. 3:**
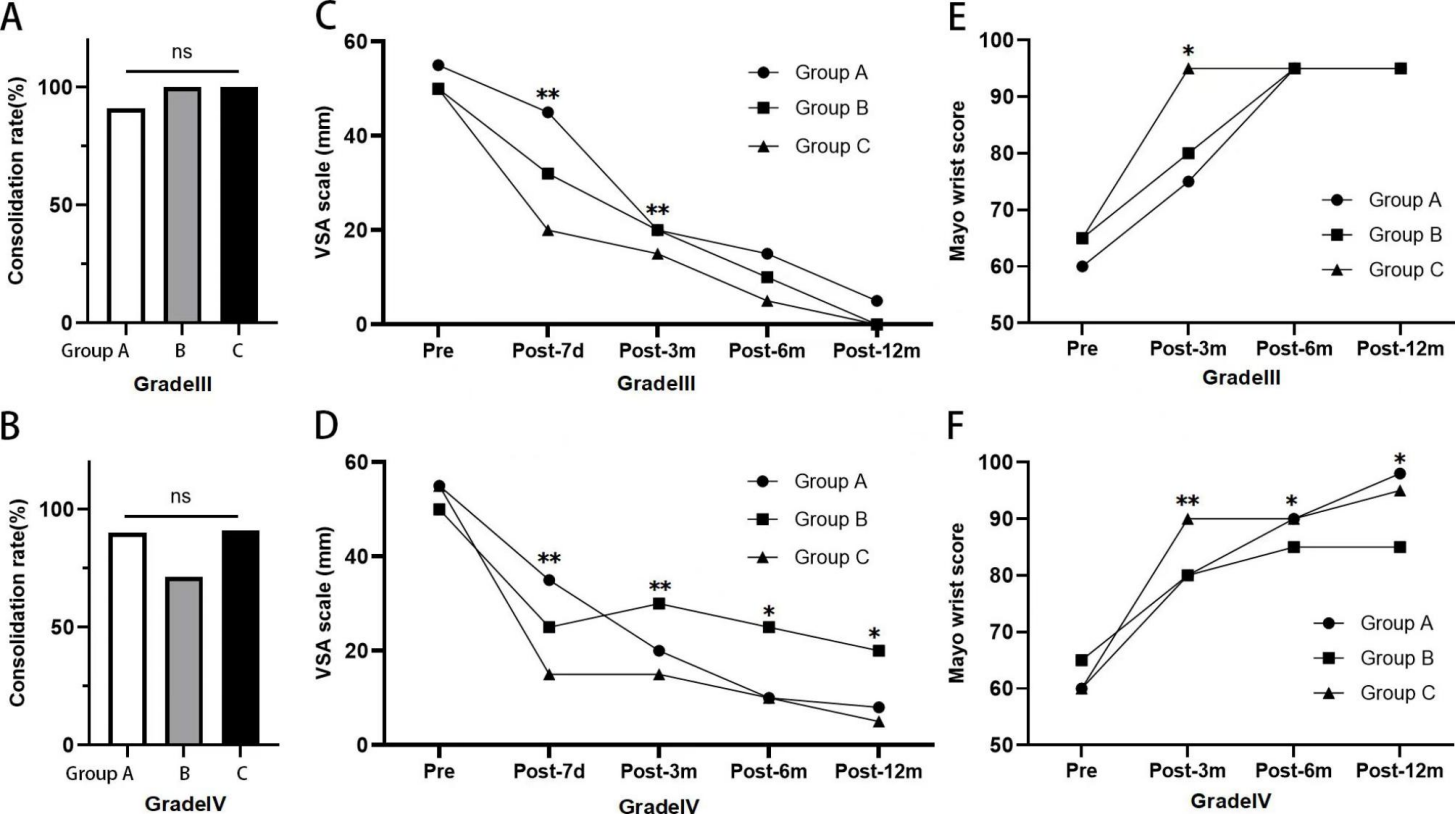
A-B. Consolidation rate of the three surgical methods for patients with Slade and Dodds Grade III and IV SNU. Group A, open surgery debridement, bone grafting, and internal fixation; Group B, percutaneous Screw Fixation; Group C, percutaneous screw fixation and PRP injection. ns = not statistically significant. Figure 3 C-F. Pre- and post-operative VSA and Mayo wrist score of the three surgical methods for patients with Slade and Dodds Grade III and IV SUN. *, p<0.05, **, p<0.01; Pre = Preoperative, Post-7d = Post- operative 7 days, Post-3 m = Post- operative 3 months, Post-6 m = Post- operative 6 months, Post-12 m = Post- operative 12 months

There was no significant difference in the consolidation rate among the three surgical groups in patients with Grade III SNU (*p =* 0.52). In patients with Grade IV SNU, the consolidation rate was lower in group B than that in group A and group C, though these differences were not significantly different (*p =* 0.46) (Table 2) (Fig. 3A-B.).

**Table 2:** Pre- and post-operative clinical outcomes of the three surgical methods for patients

Nonunion Grade	Grade III				Grade IV			
Item	Group A	Group B	Group C	*P-*value	Group A	Group B	Group C	*P-*value
Consolidation rate	90.91%	100%	100%	0.52	90.00%	71.43%	90.91%	0.46
VSA scale (mm)								
Preoperative	55(40 to 60)	50(45 to 60)	50(40 to 60)	0.36	55(40 to 60)	50(40 to 67)	55(45 to 60)	0.94
Post-operative								
7 days	45(30 to 55)^bc^	32(25 to 45)^ac^	20(10 to 30)^ab^	<0.01	35(25 to 50)^bc^	25(20 to 40)^ac^	15(10 to 20)^ab^	<0.01
3 months	20(15 to 30)^c^	20(10 to 25)	15(0 to 15)^a^	<0.01	20 (10 to 30)^c^	35(20 to 30)^c^	15(10 to 30)^ab^	<0.01
6 months	15(0 to 30)	10(5 to 20)	5(0 to 15)	0.17	10(0 to 30)^b^	25(15 to 30)^ac^	10(0 to 30)^b^	0.01
12 months	5(0 to 30)	0(0 to 15)	0(0 to 10)	0.35	8(0 to 30)^b^	20(10 to 30)^ac^	5(0 to 30)^b^	0.01
Mayo wrist score								
Preoperative	60(50 to 70)	65(60 to 70)	65(50 to 70)	0.55	60(50 to 70)	65(50 to 65)	60(50 to 65)	0.27
Post-operative								
3 months	75(70 to 95)^c^	80(80 to 85)^c^	95(80 to 95)^ab^	0.01	80(70 to 85)^c^	80(65 to 85)^c^	90(70 to 95)^ab^	<0.01
6 months	95(75 to 100)	95(90 to 100)	95(90 to 100)	0.18	90(70 to 95)^b^	85(65 to 85)^ac^	90(70 to 95)^b^	0.01
12 months	95(75 to 100)	95(90 to 100)	95(90 to 100)	0.18	98(70 to 100)^b^	85(65 to 95)^ac^	95(70 to 100)^b^	0.02

**a**. Compared with group A, *p*<0.05; **b**. Compared with group B, *p*<0.05; **c**. Compared with group C, *p*<0.05; Group A, open surgery debridement, bone grafting, and internal fixation; Group B, percutaneous Screw Fixation; Group C,percutaneous screw fixation and PRP injection

Regarding pain and wrist function, in patients with Grade III SNU, at 7 days after surgery, the VAS score was significantly lower in group C than in group A (*p <* 0.01) or group B (*p <* 0.01); the VAS score was also significantly lower in group B than in group A (*p =* 0.02). At 3 months, the VAS score was significantly lower in group C than in group A (*p <* 0.01), and the Mayo wrist score was significantly higher in group C than in groups A (*p <* 0.01) or B (*p =* 0.03). However, there were no significant differences in VAS and Mayo scores among the three groups at 6 months or 12 months. In patients with Grade IV SNU, at 7 days after surgery, the VAS score was significantly lower in group C than in group A (*p <* 0.01) and group B (*p <* 0.01); the VAS score was significantly lower in group B than in group A (*p =* 0.02). At 3 months, the VAS score was significantly lower in group C than in group A (*p <* 0.01) or group B (*p <* 0.01), and the Mayo wrist score was significantly higher in group C than in group A (*p <* 0.01) or group B (*p <* 0.01). At 6 months and 12 months, the VAS score was significantly lower, and the Mayo wrist score was significantly higher in group A and group C than in group B (P < 0.05). The VAS and Mayo scores of group A and group C were not significantly different at 6 months or 12 months after surgery (Table 2) (Fig. 3C-F.).

## Discussion

### Percutaneous screw fixation and PRP injection may achieve the same outcomes as open surgery with bone grafting in Slade and Dodds Grade IV SNU

At present, most guidelines and expert consensus statements suggest that percutaneous screw fixation is the optimal treatment strategy for Slade and Dodds Grade I-III SNU whereas Grade V-VI SNU require open debridement and bone grafting in addition to fixation, and the most appropriate treatment for Grade IV SNU remains controversial [9, 12, 13]. The treatment of the fracture ends and establishment of a microenvironment that promotes fracture healing are the main controversies at present.

The advantage of open surgery combined with bone grafting is that debridement and reduction of the fracture can be directly visualized. Bone grafting can be used to construct bone ingrowth medium and the pedicled tissue can promote neovascularization and establishment of an additional blood supply, which is widely applicable to all grades of nonunion, especially Grade V—VI SNU [14]. However, some surgeons argue that the sclerotic bone at the fracture ends does not necessarily require open debridement, percutaneous reaming is sufficient to clear sclerotic bone [15–18]. According to Mahmoud and Koptan, extensive bone resorption at the fracture ends is not an absolute indication for bone grafting. The fracture end of the nonunion is wrapped by a “cap-like structure” formed by fibrous tissue or cartilage, which stabilizes the local area and concentrates growth factors [9]. Capo et al. even demonstrated that Grade IV SNU could be treated by percutaneous nail fixation alone without supplemental bone grafting [8]. Although pedicled tissue can promote the formation of additional blood vessels, it is doubtful whether pedicled tissue can provide a stable blood supply; moreover, the original blood supply may be destroyed by the incision. In contrast, PRP is a supraphysiological concentration of platelets, rich in growth factors and other cytokines that promote cell proliferation and bone tissue healing [19]. PRP has been proved to improve the healing rate of SNU in open surgery [1, 11, 20]. Base on this finding, we speculate that PRP may expand the indications of percutaneous screw fixation for the treatment of Grade IV SNU.

Our retrospective study showed that all three types of surgery led to similarly high union healing rates for Grade III SNU, and most cases had healed at 3 months after surgery. For Grade IV SNU, the healing rate was higher in group C and group A than in group B, though the differences were not statistically significant. During follow-up, we observe that three patients with a Grade IV SNU treated by percutaneous screw fixation alone (Group B) exhibited no obvious signs of bone healing at 3 months after surgery, unlike other groups. one patient was lost to follow-up, and two patients were excluded from the analysis as they underwent subsequent surgery within 6 months. As a result, we rarely conducted percutaneous screw fixation alone on subsequent patients with Grade IV SNU, prompting us to explore the feasibility of PRP to strengthen percutaneous screw fixation for Grade IV SNU. In general, the results of this study are consistent with the conclusions of previous studies that reported percutaneous fixation is a suitable treatment for Grade I-III SNU [4–9]. However, caution should be exercised when using percutaneous treatment alone for Grade IV SNU, as its therapeutic effects were still lower than open surgery with bone grafting and internal fixation, although not statistically significant. The addition of PRP (group C) may account for the improvement of percutaneous Screw Fixation alone (group B), even led to similar outcomes as incision (group A). Therefore, we suggest that percutaneous screw fixation combined with PRP injection may be an effective alternative to incision for the treatment of Grade IV SNU.

### Treatment of SNU with percutaneous screw fixation and PRP injection is likely to reduce early postoperative pain and promotes the recovery of wrist function

As most patients with SNU are young, bone healing is only the basic goal of treatment; effective, rapid recovery of wrist function is the ultimate treatment goal. In addition, in our previous clinical experience, many patients still report long-term pain and wrist movement disorders after surgery that significantly adversely affect their life and work. Therefore, orthopedic surgeons seek a new therapeutic technique to promote the recovery of wrist function.

PRP is widely used and achieves good results in the treatment of osteochondral injury and various types of arthritis [21, 22]. Namazi et al. showed that injection of PRP in patients with Herbert B2 scaphoid fractures treated with plaster immobilization significantly reduced pain at rest and improved wrist function scores [2]. Moreover, De Vitis R et al. find that PRP may improve wrist function, and relieve pain after 3 months from open surgery [1]. However, there have been no previous studies of the effect of PRP on pain and function in patient received percutaneous screw fixation.

In this study, in both Grade III and Grade IV SNU, group C had significantly better Mayo scores and VAS scores than group A or group B in the early stages after surgery (3 months). This indicates that the effects of PRP—in terms of reduced pain and improved joint function—are mainly sustained in the first 3 months. Interestingly, in both Grade III and Grade IV SNU, some patients treated with open surgery debridement, bone grafting, and internal fixation (group A) who were radiologically diagnosed with bone healing still reported obvious wrist pain and limited joint activity, although their scores were better than those of patients with a nonunion that did not heal radiologically. We speculate that joint capsule damage or scar contracture may cause this pain and limit joint function. Therefore, we conclude that percutaneous screw fixation with PRP injection may be a superior alternative to incision for the treatment of Grade III and Grade IV SNU.

### Limitations

This was a single-center retrospective study with a small sample size, the measurement indicators may be affected by the patients’ subjective opinions, and the average follow-up duration was 1 year. Thus, additional analyses of prospective large cohort treated at multiple centers with longer follow-up periods are necessary to confirm the conclusions of this paper. The effect of PRP in patients except Grade III and IV SNU needs further demonstrated.

## Electronic supplementary material

Below is the link to the electronic supplementary material.

Supplementary Material 1

## Data Availability

The datasets used or analyzed during the current study are available from the corresponding author on reasonable request.
