# Supplementary material for: Comparative effectiveness of three treatment options for slade and dodds grade III-IV scaphoid nonunion: a retrospective study
Source: BMC Musculoskelet Disord. 2023 Mar 17;24:204. doi: 10.1186/s12891-023-06320-1 (PMC10022066; doi:10.1186/s12891-023-06320-1)
Supplement: Supplementary file 1 — Supplementary Material 1 [file 12891_2023_6320_MOESM1_ESM.doc]

**Appendix**

| Appendix Table 1: Classification System for Scaphoid Nonunions | | |
| --- | --- | --- |
| Grade | Category | Characteristics of scaphoid nonunions |
| I | Delayed presentation | Scaphoid fractures with delayed presentation (4–8 weeks) |
| II | Fibrous nonunion | Intact cartilaginous envelope, minimal fracture line at nonunion interface, no cyst or sclerosis |
| III | Minimal sclerosis | < 1 mm bone resorption at nonunion interface, minimal sclerosis |
| IV | Cyst formation and sclerosis | < 5 mm bone resorption at nonunion interface, cyst formation, scaphoid alignment maintained |
| V | Cyst formation and sclerosis | 5—10 mm bone resorption at nonunion interface, cyst formation, and maintained scaphoid alignment maintained |
| VI | Pseudarthrosis | Separate bone fracture fragments with profound bone resorption at nonunion interface; gross fragment motion and deformity often present |
